# CDK4/6 inhibitors in hormone receptor-positive, human epidermal growth factor receptor 2 (HER2)-negative metastatic breast cancer: Are we at the finish line?

**DOI:** 10.18632/oncotarget.26134

**Published:** 2018-09-28

**Authors:** Clinton Yam, Mien-Chie Hung, Gabriel N. Hortobagyi

**Affiliations:** Department of Breast Medical Oncology, The University of Texas MD Anderson Cancer Center, Houston, TX, USA

**Keywords:** CDK4/6 inhibitors, hormone receptor-positive, metastatic breast cancer

Small-molecule inhibitors of cyclin-dependent kinases 4 and 6 (CDK4/6) have transformed the treatment landscape of hormone receptor (HR)-positive and human epidermal growth factor receptor 2 (HER2)-negative metastatic breast cancer, leading to significantly improved outcomes for patients. When used in combination with aromatase inhibitors in the frontline setting for postmenopausal patients with HR-positive, HER2-negative metastatic breast cancer, CDK4/6 inhibitors have led to significant improvements in progression-free survival (PFS), with hazard ratios ranging from 0.54 to 0.58 [1-3].

Mechanistically, CDK4/6 inhibitors interfere with the interaction between CDK4/6 and cyclin D, preventing the hyperphosphorylation of the retinoblastoma (Rb) gene product, thereby halting progression through the G1 checkpoint to the S phase of the cell cycle [4, 5].

There are currently three CDK4/6 inhibitors approved by the US Food and Drug Administration (FDA) (ribociclib, palbociclib, and abemaciclib) in combination with endocrine therapy for frontline therapy for HR-positive, HER2-negative metastatic breast cancer in postmenopausal women and while all seem to benefit a broad patient population according to subset analyses published along with the primary results of the respective registration trials, objective response rates were reported to be between 40.7-48.2% among all patients and 52.7-59.2% among patients with measurable disease [1-3], suggesting that 40-60% of patients could potentially benefit from novel combinatorial approaches targeting primary resistance to CDK4/6 inhibitors.

Updated efficacy and safety data from the second interim analysis of the Mammary Oncology Assessment of LEE011’s (ribociclib’s) Efficacy and Safety (MONALEESA-2) trial were recently published along with results of exploratory biomarker analyses [6]. Briefly, MONALEESA-2 was a randomized, double-blind, placebo-controlled, phase III trial [1] in which a total of 668 postmenopausal women with HR-positive, HER2-negative metastatic breast cancer who had not previously received systemic therapy for metastatic disease were randomly assigned to receive oral ribociclib plus letrozole (n = 334) or placebo plus letrozole (n = 334). At the first interim analysis, the median PFS for the ribociclib group was not reached while that of the placebo group was 14.7 months (hazard ratio: 0.56; 95% CI: 0.43-0.72; p = 3.29 x 10^-6^) [1]. In this updated analysis, with a median duration of follow-up of 26.4 months, the median PFS for the ribociclib and placebo groups were 25.3 and 16.0 months, respectively (hazard ratio: 0.568; 95% CI: 0.457-0.704, log-rank p = 9.63 x 10^-8^), and overall survival (OS) data remained immature [6]. Safety data at the second interim analysis remained similar to those reported with the initial publication [1], with no new, unexpected or cumulative toxicities [6].

Early results of correlative studies from MONALEESA-2 were also reported along with the updated clinical data [6]. Baseline circulating tumor DNA (ctDNA) samples were sequenced in 74% (494/668) of patients. No genetic alterations were detected in baseline samples from 67 patients, leaving samples from 427 patients with adequate genomic data for correlation with clinical outcomes. Short variants (mutations and short insertions/indels) of *PIK3CA* and *TP53* were detected in 33% (142/427) and 12% (53/427) of patients, respectively. Receptor tyrosine kinase (RTK) signaling pathway genes were found to be altered (amplifications and short variants) in 12% (51/427) of patients. PFS benefit with ribociclib was observed regardless of *PIK3CA* or *TP53* mutation status (Table 1). However, patients with altered RTK signaling pathway genes were found to derive less benefit from the addition of ribociclib to letrozole, compared to patients with wild type RTK signaling pathway genes (Table 1).

**Table 1:** Effect of alterations in *PIK3CA*, *TP53* and receptor tyrosine kinase (RTK) signaling pathway genes on progression-free survival (PFS) in patients receiving ribociclib or placebo in combination with letrozole

	*PIK3CA*			
	Wild type		Altered	
	Ribociclib + Letrozole (n=143)	Placebo + Letrozole (n=142)	Ribociclib + Letrozole (n=69)	Placebo + Letrozole (n=73)
PFS events, n	54	93	40	55
Median PFS, months (95% CI)	29.6 (24.84-NR)	14.69 (13.04-19.15)	19.15 (13.01-23.85)	12.71 (9.23-14.98)
Hazard ratio (95% CI)	0.44 (0.31-0.62)		0.53 (0.35-0.81)	
	***TP53***			
	**Wild type**		**Altered**	
	Ribociclib + Letrozole (n=180)	Placebo + Letrozole (n=194)	Ribociclib + Letrozole (n=32)	Placebo + Letrozole (n=21)
PFS events, n	72	129	22	19
Median PFS, months (95% CI)	27.63 (24.61-30.92)	14.69 (13.04-16.72)	10.22 (5.39-22.14)	5.52 (1.84-7.39)
Hazard ratio (95% CI)	0.44 (0.33-0.59)		0.43 (0.23-0.83)	
	**RTK signaling pathway genes**			
	**Wild type**		**Altered**	
	Ribociclib + Letrozole (n=189)	Placebo + Letrozole (n=187)	Ribociclib + Letrozole (n=23)	Placebo + Letrozole (n=28)
PFS events, n	81	128	13	20
Median PFS, months (95% CI)	24.84 (22.21-30.92)	14.39 (12.85-16.46)	21.29 (5.52-NR)	11.43 (9.07-19.15)
Hazard ratio (95% CI)	0.46 (0.35-0.62)		0.73 (0.35-1.54)	

Abbreviations

1) PFS: Progression-Free Survival

2) CI: Confidence Interval

3) RTK: Receptor Tyrosine Kinase

The updated efficacy and safety data from MONALEESA-2 reported in this study provide strong evidence in support of the therapeutic benefit and tolerability of ribociclib in combination with letrozole for frontline therapy of HR-positive, HER2-negative metastatic breast cancer. The failure of *PIK3CA* mutation status as a predictive biomarker is consistent with what was observed in PALOMA-3 [7, 8]. However, it has been reported that early declines in *PIK3CA* ctDNA levels are associated with improved PFS in patients treated with palbociclib and fulvestrant in PALOMA-3, suggesting that dynamic changes in the genomic landscape of the tumors may be more informative than baseline genomic alterations alone [8]. While exploratory, the observation that patients with altered RTK signaling pathway genes seemed to derive less benefit from the addition of ribociclib to letrozole suggest that such pathways may represent mechanisms of resistance to CDK4/6 inhibitors, laying the ground for future preclinical and clinical studies evaluating novel combinatorial approaches involving CDK4/6 and RTK inhibitors.

Although CDK4/6 inhibitors have ushered in a new treatment paradigm for HR-positive, HER2-negative metastatic breast cancer, several questions remain unresolved. First, it is unclear if patients will benefit from continued CDK4/6 inhibition following progression on a frontline CDK4/6 and aromatase inhibitor combination and the results of several ongoing randomized trials should provide an answer to this question (NCT02632045, NCT02732119). Second, since fulvestrant has demonstrated superior efficacy as a single agent compared to anastrazole for the frontline treatment of advanced HR-positive breast cancer [9], it would be important to determine whether this difference in efficacy is maintained when used combination with CDK4/6 inhibitors. This issue is especially important in light of the recent approval of ribociclib in combination with fulvestrant in the frontline setting based on data from MONALEESA-3 [10]. Third, because CDK4/6 inhibitors have been approved in both the first and second line settings for patients with HR-positive, HER2-negative metastatic breast cancer, whether CDK4/6 inhibitors should be used upfront or reserved for the second line setting remains a major clinical dilemma and the randomized, phase III SONIA study aims to answer this question (NCT03425838). Fourth, there is limited data on clinically relevant mechanisms of primary and acquired resistance to CDK4/6 inhibitors as well as the existence and extent of cross-resistance between the three currently available CDK4/6 inhibitors. While the respective phase III studies [1-3] suggest that presently available CDK4/6 inhibitors are virtually identical in therapeutic activity and only modestly differ in safety profile, no studies have compared these agents directly, resulting in a lack of data to help inform clinical practice. Thus, additional correlative studies from completed randomized phase III studies [1-3] as well as ongoing biomarker studies incorporating multi-omics analyses of tumor tissue at baseline and progression (NCT03050398, NCT03195192) will be instrumental in guiding future studies aimed at maximizing response and overcoming resistance to these agents. In addition to the setting for which they are currently approved, CDK4/6 inhibitors are also being tested in the adjuvant (NCT03285412, NCT03078751) and neoadjuvant settings (NCT02712723, NCT03248427) as well as in other subtypes of breast cancer such as triple-negative (NCT03090165, NCT03130439) and HER2-positive disease (NCT02657343, NCT03054363). We await with great interest the results of these studies and the OS data from the various phase III trials [1-3].

In summary, the success of CDK4/6 inhibitors has moved the field forward significantly and, more importantly, improved the lives of patients with HR-positive, HER2-negative metastatic breast cancer. However, there is still much we have to learn about these agents to maximize their clinical efficacy and additional data from completed and ongoing trials will certainly provide greater clarity as we continue to strive to improve outcomes for our patients.
